# Deep Learning Model for Osteoporosis Screening From Chest Radiographs: A Multicenter Analysis of External Robustness and Model Calibration

**DOI:** 10.7759/cureus.89446

**Published:** 2025-08-05

**Authors:** Yuichi Imoto, Takahiro Inui, Kentaro Matsui, Yoshinobu Watanabe, Takashi Igari, Shu Takeuchi, Mana Kimura, Satoshi Yagi, Hirotaka Kawano

**Affiliations:** 1 Department of Orthopaedic Surgery, Teikyo University School of Medicine, Tokyo, JPN; 2 Trauma and Reconstruction Center, Teikyo University Hospital, Tokyo, JPN; 3 AI Integration Section, Technology Administration Supervision Department, Fuji Soft Incorporated, Tokyo, JPN

**Keywords:** artificial intelligence, calibration, chest radiographs, chest x-ray (cx-ray), deep learning, external robustness, external validation, osteoporosis screening

## Abstract

Osteoporosis is a common condition, and treatment can reduce the risk of fracture and extend healthy life expectancy, but most cases go undiagnosed and untreated. Dual-energy X-ray absorptiometry (DXA), the gold standard for diagnosing osteoporosis, is costly, time-consuming, and labor-intensive, with limited availability in low-resource settings and small clinics, so it is not suitable for screening for potential osteoporosis. To address this problem, in recent years, some studies have attempted to screen for osteoporosis by estimating DXA bone mineral density (BMD) from chest radiographs (CR), which are frequently used in daily clinical practice, by applying deep learning technology. Although these models have shown good screening performance, the performance of the external data from different facilities and equipments still requires further investigation. In this study, we developed deep learning models for osteoporosis screening and determined the performance of internal and external data. The performance on internal data was good across all models, accurately predicting osteoporosis diagnosed by DXA. Performance on external data exceeded that of calcaneal quantitative ultrasound (QUS), which is widely used as a screening tool for osteoporosis. The screening performance for external data was poor compared to internal data, but by mixing at least 500 external data into the training data, the model could be calibrated and the performance improved. Our results demonstrate that the model can easily perform osteoporosis screening from CR, the most commonly performed imaging test worldwide, without additional invasiveness or cost.

## Introduction

Osteoporosis has become a major health concern worldwide as life expectancy increases and the population continues to age. Approximately 500 million people worldwide have osteoporosis [1], and one in three women and one in five men above 50 years of age will experience fragility fractures during their lifetime [2] because of osteoporosis. Fragility fractures significantly impact the patients' activities of daily living (ADL) and prognosis, cause death, and increase disability-adjusted life years (DALYs) [3], resulting in a significant financial burden on the national health insurance system [4]. Therefore, osteoporosis should be diagnosed and treated early to prevent fragility fractures. However, several patients remain untreated because osteoporosis is asymptomatic until it causes fragility fractures [4], which is attributed to inadequate screening for osteoporosis.

Dual-energy X-ray absorptiometry (DXA), while the gold standard [5,6], is costly with an acquisition cost of approximately €45,000 [7] and requires trained personnel [8]. It is not commonly available in small clinics and low-resource settings and is used solely for bone density assessment, limiting its broader clinical utility. Additionally, DXA is an independent test that measures bone mineral density (BMD) only, without screening for multiple diseases simultaneously, such as chest radiograph (CR) or blood tests, limiting the opportunity for osteoporosis diagnosis [9]. Although calcaneal quantitative ultrasound (QUS), which is inexpensive and noninvasive without the risk of radiation exposure [10], is sometimes used as a screening tool [11,12], it is not recommended for definitive diagnosis because of its large error rate compared with DXA [13]. Screening examinations for early diagnosis of osteoporosis require using specific equipment; however, they have poor performance. Therefore, a simpler and more effective diagnostic tool is required.

Recently, innovative studies attempting to diagnose osteoporosis using CR as an alternative screening tool [14,15] by applying artificial intelligence technologies, including deep learning, have been reported. However, reports on the external validity of these models remain sparse. Although a recent multicenter study has attempted external validation of deep learning models for BMD prediction from CR [16], the specifics concerning imaging equipment and other acquisition parameters are not fully elucidated. Such incomplete elucidation can potentially limit the true generalizability of these models; indeed, the inadvertent inclusion of images captured by similar equipment in both development and validation datasets may lead to an overestimation of external robustness. Therefore, a more comprehensive establishment of external validity necessitates further investigation. Furthermore, although some studies have claimed that diagnostic deep learning-based AIs have excellent diagnostic capabilities, comparable to those of specialist physicians [17-19], they hide significant biases and poor robustness in external facilities [20,21]. The robustness of external data is crucial in medical AI [22], particularly when using examinations conducted in various settings, such as CR, and should be verified [23-26]. It is also important to clarify the amount of data needed for calibration to adapt the model to an external facility if performance is degraded [20,21]. The primary aim of this study was to clarify the internal and external performance of AI models for estimating BMD using CR from a multicenter dataset. Secondary aims included investigating the impact of adding external data to the training set on model performance and elucidating the robustness and feasibility of model calibration for clinical implementation in external facilities.

## Materials and methods

This retrospective study was conducted in accordance with the principles of the Declaration of Helsinki and current scientific guidelines. The Teikyo University Ethical Review Board for Medical and Health Research Involving Human Subjects approved the study protocol (approval number 19-152). Informed consent was waived by the Teikyo University Ethical Review Board. Participant consent was obtained based on the opt-out method. The performance of the model was verified using a checklist for AI in medical imaging (CLAIM) [27].

Study population

This study used data from adult patients who visited the orthopedics or health screening centers of Teikyo University-affiliated facilities (Ageo Central General Facility and Misato Central General Facility) between January 2000 and July 2019. The inclusion criteria were adult patients who underwent plain CR and DXA within two months before or after the examination date. The exclusion criteria were CRs of poor quality and images containing embedded personal information, such as names. This study included data from 9725 cases (4485 Ageo and 4319 Misato), excluding 28 cases with embedded personal information. For CR acquisition, Ageo Central General Facility utilized Shimadzu RAD Speed Pro and UD-150B-30 X-ray units with FUJIFILM CALNEO series Flat Panel Detectors (FPDs). Misato Central General Facility employed Shimadzu RAD Speed Pro and UD-150B-40 X-ray units with Konica Minolta Aero DR FPDs. CRs were acquired in various locations, such as health screening centers, outpatient clinics, and operating rooms; therefore, image brightness, background, and other imaging conditions were not uniform, resulting in a diverse dataset.

**Figure 1:**
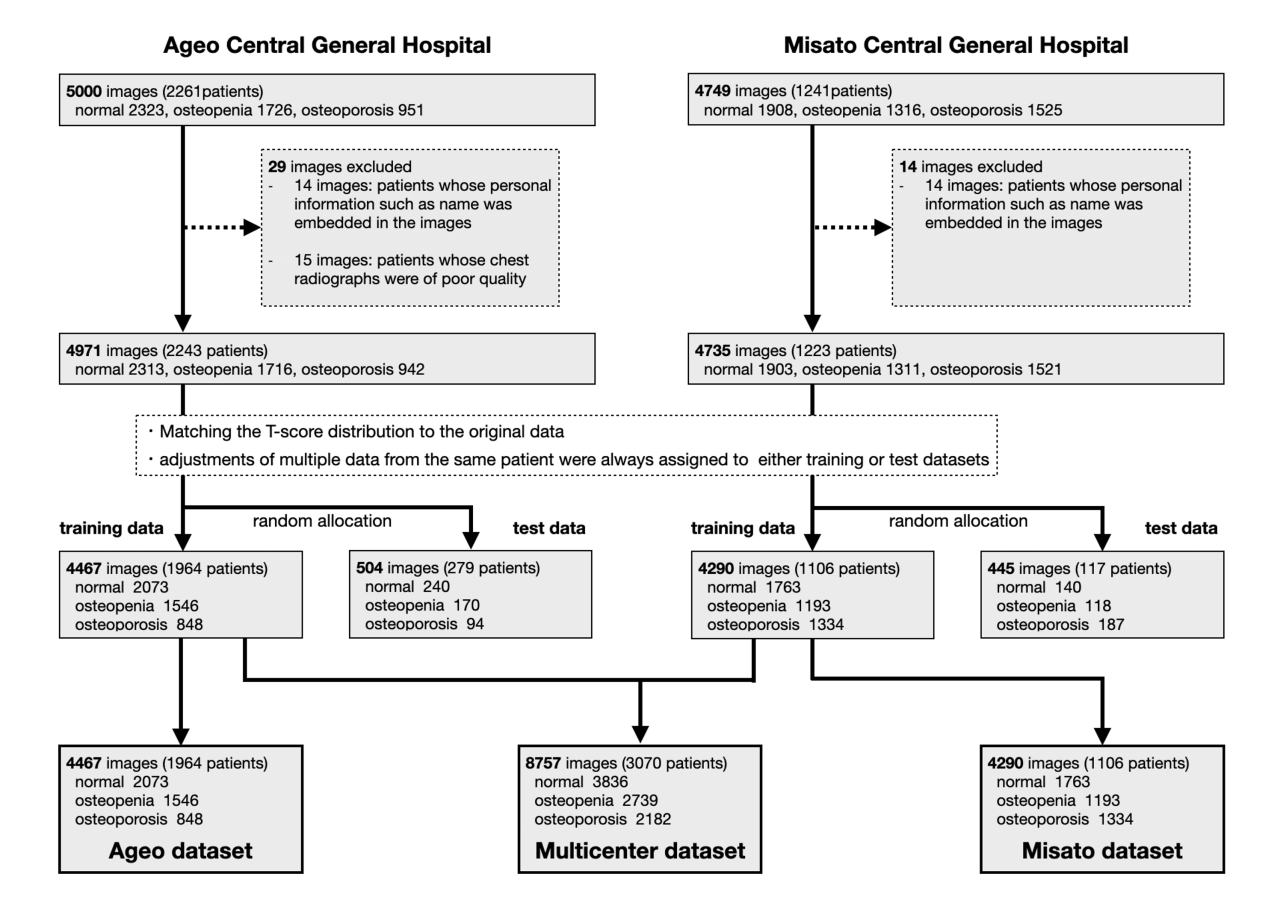
Flowchart for an assortment of datasets

This study paired T-scores calculated from CRs and DXA examinations (Misato: HOLOGIC Discovery Ci; Ageo: GE PRODIGY C) conducted within two months before or after the CR examination date. BMD measurements and their quality assurance were performed by trained radiological technologists at each facility following standard clinical protocols, and T-scores were automatically generated by the DXA equipment. DXA was performed at the lumbar spine or femoral neck; when both sites were assessed, the lower score was used (5863 femoral and 3843 lumbar). Participants were classified into three groups based on the WHO criteria for osteoporosis: normal (T-score ≥-1.0), osteopenia (-1.0 > T-score > -2.5), and osteoporosis (T-score ≤-2.5) [28]. These data were randomly assigned to training (90%) and test (10%) datasets using the model-selection module in scikit-learn to ensure that the T-score distribution matched that of the overall dataset. For model validation, 10% of the training dataset was randomly selected for each training session. Additionally, care was taken to ensure that multiple data points from the same patient were assigned exclusively to either the training or the test dataset. For cross-facility evaluation, data from one facility were used for training, and those from the other facility were used for testing (Figure 1).

Experimental setup

For image preprocessing, CRs in DICOM format were first converted to 8-bit RGB (three-channel) JPEG images without altering the window level. The images were then padded with a black background to form squares and resized to 224×224 pixels. Image normalization was also performed.

Deep learning model

Based on the Python (version 3.6.9) programming language, we used the machine learning framework PyTorch (version 1.7.1) [29] and the deep learning library fast.ai (version 1.0.60) [30] to train a deep learning model called EfficientNetV2 (B4) [31] using the NVIDIA Tesla V100 graphics processing unit. EfficientNetV2 is a recent image recognition model used for diagnostic purposes and is known for its excellent recognition capabilities. We selected the B4 version because it offers a good balance of performance and shorter training time due to fewer parameters compared to other EfficientNetV2 models. According to previous reports, fine-tuning offers better performance for medical images than training a deep learning model from scratch [32]. This study used pre-trained weights from the ImageNet dataset [33], which contains 1000 classes. Fine-tuning was performed using the CRs obtained in this study. Because the CRs were consistently captured in the same direction, data augmentation was not performed, and the dataset was not expanded. We used the 1cycle policy optimizer from the fast.ai package and trained the model for 100 epochs with a maximum learning rate of 0.001. The optimizer used was Adam, and the batch size was 64, consistent with the default settings of the fast.ai 1cycle policy unless otherwise specified. Additionally, the fast.ai package was used to split the training and validation datasets in an 80:20 ratio for model training.

The sigmoid function was used as the activation function, and binary classification was performed using values ranging from 0 to 1 in the output layer with a set threshold value. The optimal threshold for the validation data (internal test data) was determined using the point on the ROC curve closest to (0,1) [34], and this threshold was applied to the model.

Two models were developed using the above learning process: one with a T-score cutoff of -2.5 (T-2.5 model) and another with a cutoff of -1.0 (T-1.0 model).

Assessment

Performance of the Deep-Learning Model

To evaluate diagnostic performance against the validation dataset, accuracy, sensitivity, specificity, and area under the ROC curve were calculated. ROC curves were plotted for sensitivity versus 1-specificity using scikit-learn and matplotlib, and the area under the curve (AUC) was compared using DeLong’s algorithm [35,36]. The confusion matrix in this study was presented as a 2×2 contingency table, displaying the numbers of true positives, false positives, true negatives, and false negatives.

To evaluate the external data’s performance, test data from a different facility was used rather than that from the training facility. Multicenter models were evaluated using test data from both facilities. The t-distributed stochastic neighbor embedding (t-SNE) technique reduced the dimensions to a two-dimensional map while maintaining the distance relationship to visualize the distribution of high-dimensional features, such as embedded categories in a model. This study applied the t-SNE method to the images or models of each facility to visualize their features.

Heatmap Generation

The gradient-weighted class activation mapping (Grad-CAM) technique was used to visually illustrate the areas of the image that the screening model focused on when making decisions. This technique generates a localized heat map at the final layer of a conventional neural network, which indicates important regions in the image. The redder regions indicate more important features. This technique outputs the diagnostic rationale for each image and visualizes it overlaid on each model.

Statistical Analyses

Bootstrapping was used to calculate the 95% CI of the AUC in this study using the roc_auc_score function of scikit-learn (n_bootstraps=10000). The Wilson score interval [37] was used to calculate the 95% CIs for accuracy, sensitivity, and specificity using the Statsmodels module in Python.

## Results

Patients' characteristics

**Figure 2:**
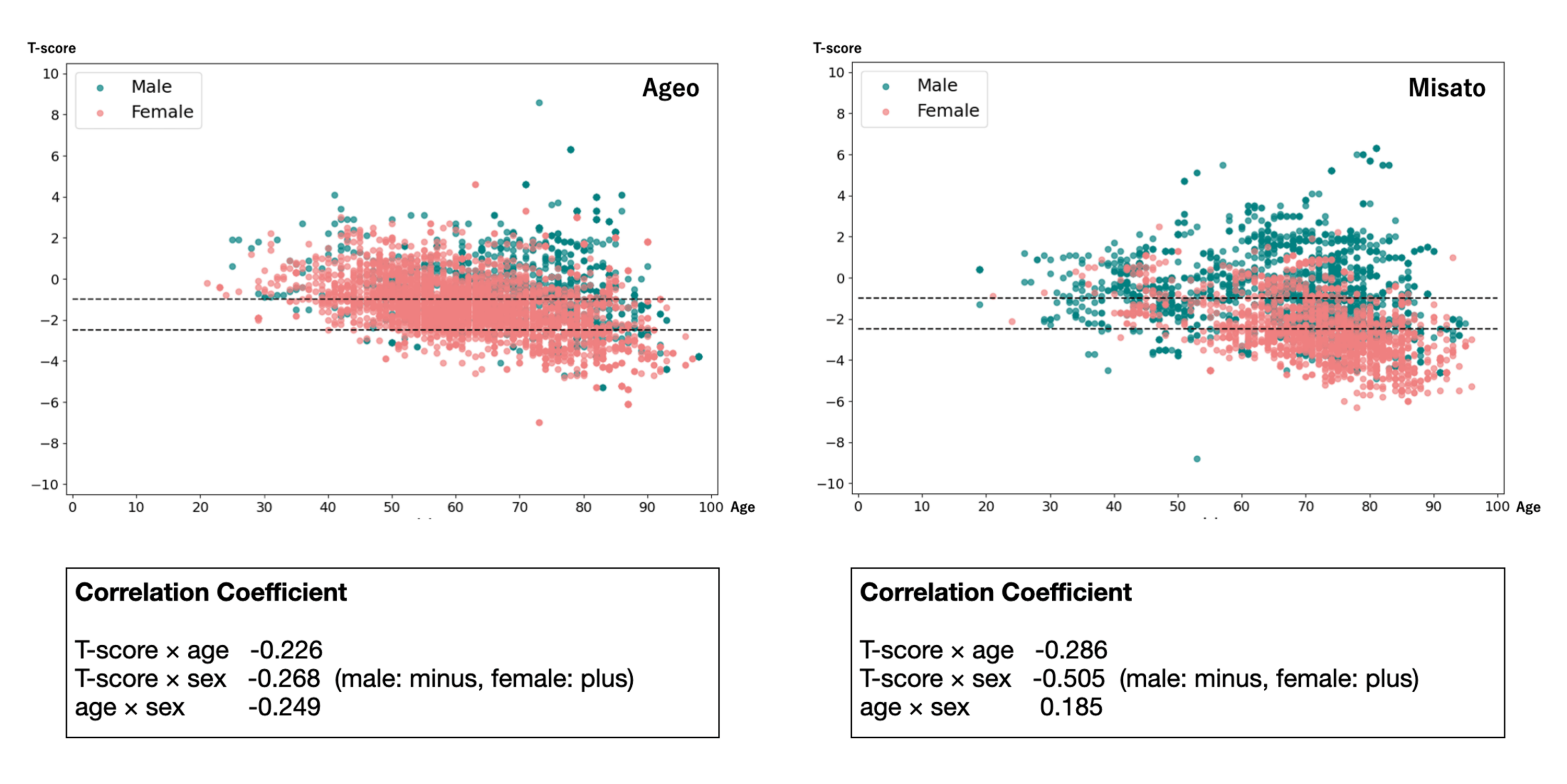
Scatter plots and correlation coefficients of patient data for each hospital The vertical axis indicates T-score, the horizontal axis indicates age, green dots indicate males, and red dots indicate females.

The study included 5199 (Ageo 3272, Misato 1699) and 4507 (Ageo 1699, Misato 2808) CRs of women and men, respectively, with ages ranging from 20 to 90 years (mean age 68.1 years). In the two months before and following the date of taking the CRs, a DXA examination found 4216, 3027, and 2463 CRs were normal, osteopenia, and osteoporosis, respectively. Table 1 shows the baseline characteristics, age, and BMD at each facility. We also plotted a scatter plot by sex using the data collected from this study, with the T-score on the vertical axis and age on the horizontal axis, to confirm that there was no significant correlation between the T-score, age, and sex at each facility (Figure 2). 

**Table 1:** Demographic characteristics of each facility BMD, bone mineral density

Characteristics	Ageo Central General Hospital			Misato Central General Hospital			Overall		
	Male	Female	All	Male	Female	All	Male	Female	All
Number of data (number of patients)	1699 (537)	3272 (1706)	4971 (2243)	2808 (455)	1927 (768)	4735 (1223)	4507 (992)	5199 (2474)	9706 (3466)
Age (years), mean±SD	71.46±11.72	64.31±13.88	66.75±13.61	67.49±13.48	72.30±11.03	69.45±12.76	68.99±12.99	67.27±13.46	68.07±13.27
T-score, mean±SD									
L1-L4	-0.33±2.16	-1.37±1.75	-0.89±2.02	0.27±1.93	-1.72±1.45	-0.52±2.01	0.10±2.02	-1.57±1.59	-0.65±2.02
Total femur	-0.50±1.48	-1.41±1.30	-1.16±1.41	-0.95±1.59	-2.92±1.26	-1.83±1.75	-0.79±1.57	-1.93±1.47	-1.47±1.61
BMD (g/cm^2^), mean±SD									
L1-L4	1.14±0.26	0.95±0.21	1.04±0.25	1.02±0.22	0.80±0.16	0.93±0.22	1.05±0.24	0.86±0.20	0.97±0.24
Total femur	0.88±0.19	0.76±0.16	0.80±0.17	0.70±0.14	0.53±0.11	0.63±0.16	0.77±0.18	0.68±0.18	0.72±0.19
Osteoporosis categories (%)									
Normal	1040 (61.21)	1273 (38.91)	2313 (46.53)	1591 (56.66)	312 (16.19)	1903 (40.19)	2631 (58.38)	1585 (30.49)	4216 (43.44)
Osteopenia	429 (25.25)	1287 (39.33)	1716 (34.52)	871 (31.02)	440 (22.83)	1311 (27.69)	1300 (28.84)	1727 (33.22)	3027 (31.19)
Osteoporosis	230 (13.54)	712 (21.76)	942 (18.95)	346 (12.32)	1175 (60.98)	1521 (32.12)	576 (12.78)	1887 (36.30)	2463 (25.38)

Overall screening model performance

**Figure 3:**
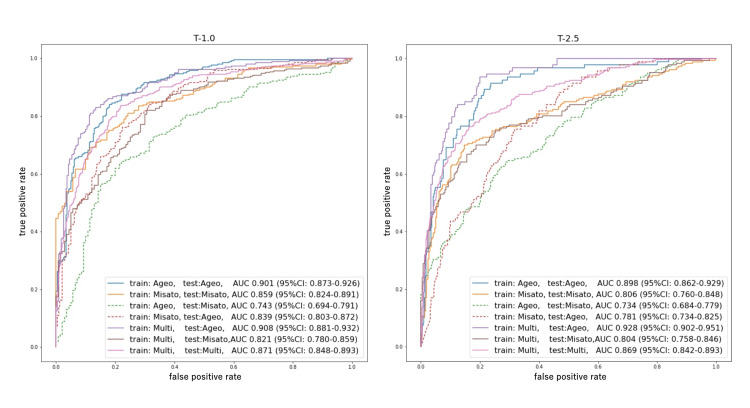
ROC curves for each model

**Figure 4:**
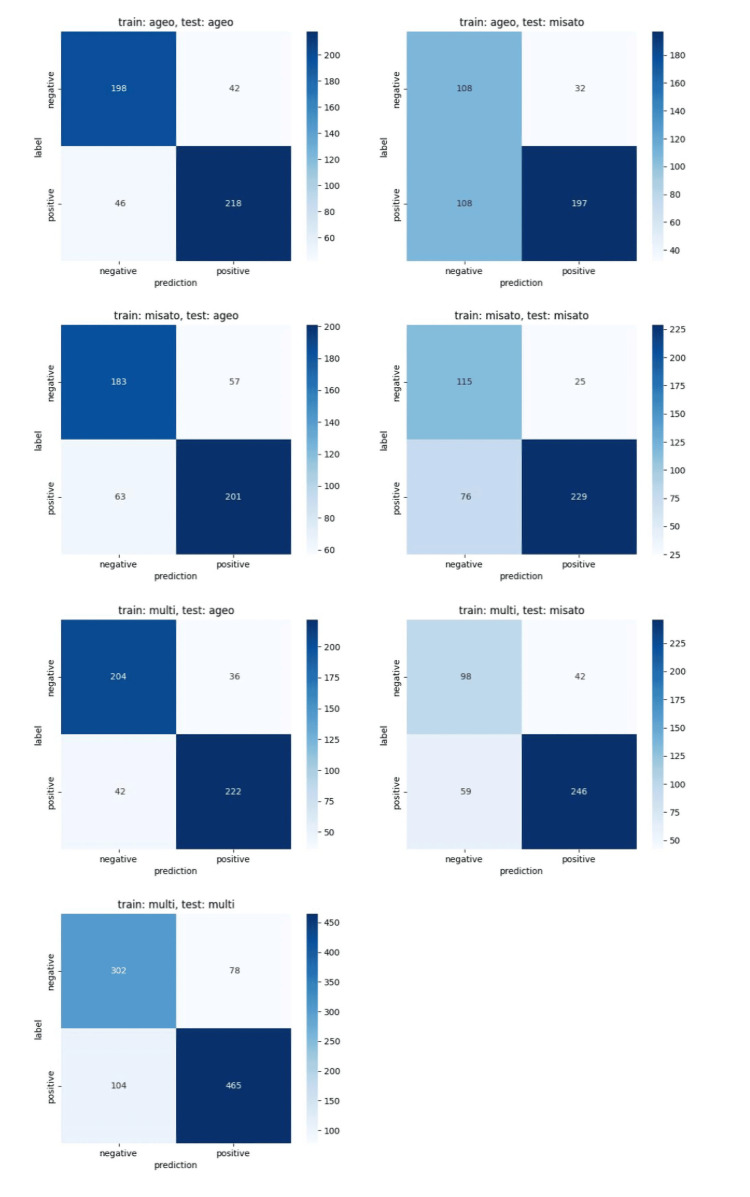
Confusion matrices of the T-1.0 model for internal and external datasets

**Figure 5:**
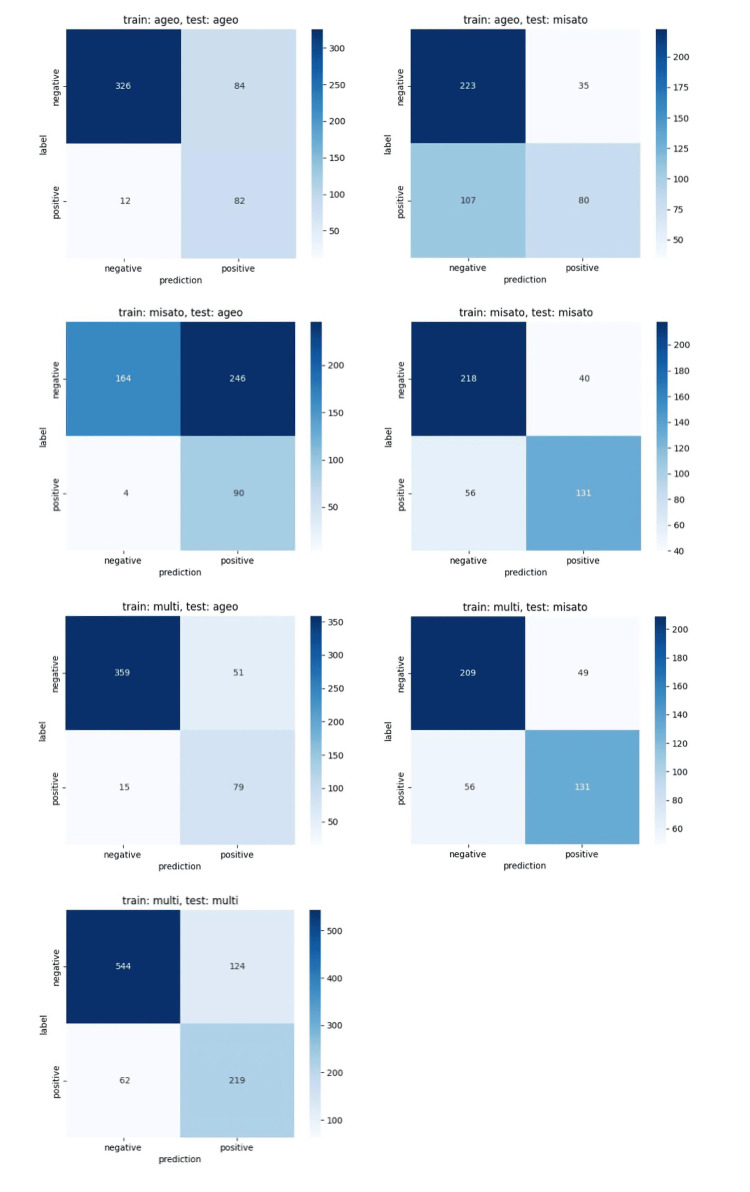
Confusion matrices of the T-2.5 model for internal and external datasets

Table 2 shows the screening performance of the developed osteoporosis screening models (Ageo, Misato, and multicenter models) against the internal and external data. Figure 3 shows the ROC curves for each model. In the multicenter model, the T-1.0 model had an AUC of 0.871 (95% CI, 0.848-0.893), sensitivity of 81.7% (95% CI, 78.3-84.7), and specificity of 0.795 (95% CI, 75.1-83.2), and the T-2.5 model had an AUC of 0.869 (95% CI, 0.842-0.893), sensitivity of 077.9 % (95% CI, 72.7-82.4), and specificity of 81.4 (95% CI, 78.3-84.2). Figure 4 and Figure 5 show the confusion metrics for each model. All models showed good diagnostic performance with an AUC of 0.80 or higher for internal test data, but the performance of all models decreased for external data (Table 2).

**Table 2:** Screening model performance for internal/external datasets Parentheses show 95% CI. AUC, area under the ROC curve

Training data	Test data	T-1.0 model				T-2.5 model			
		Sensitivity (%)	Specificity (%)	Accuracy (%)	AUC	Sensitivity (%)	Specificity (%)	Accuracy (%)	AUC
Ageo	Ageo	82.6 (77.5-86.7)	82.5 (77.2-86.8)	82.5 (79.0-85.6)	0.901 (0.873-0.926)	87.2 (79.0-92.5)	79.5 (75.3-83.1)	81.0 (77.3-84.1)	0.898 (0.862-0.929)
Ageo	Misato	64.6 (59.1-69.7)	77.1 (69.5-83.3)	68.5 (64.1-72.7)	0.743 (0.694-0.791)	42.8 (35.9-49.9)	86.4 (81.7-90.1)	68.1 (63.6-72.3)	0.734 (0.684-0.779)
Misato	Misato	75.1 (69.9-79.6)	82.1 (75.0-87.6)	77.3 (73.2-81.0)	0.859 (0.824-0.891)	70.1 (63.1-76.2)	84.5 (79.6-88.4)	78.4 (74.4-82.0)	0.806 (0.760-0.848)
Misato	Ageo	76.1 (70.6-80.9)	76.2 (70.5-81.2)	76.2 (72.3-79.7)	0.839 (0.803-0.872)	95.7 (89.6-98.3)	40.0 (35.4-44.8)	50.4 (46.0-54.7)	0.781 (0.734-0.825)
Multi	Ageo	84.1 (79.2-88.0)	85.0 (79.9-89.0)	84.5 (81.1-87.4)	0.908 (0.881-0.932)	84.0 (75.3-90.1)	87.6 (84.0-90.4)	86.9 (83.7-89.6)	0.928 (0.902-0.951)
Multi	Misato	80.7 (75.9-84.7)	70.0 (62.0-77.0)	77.3 (73.2-81.0)	0.821 (0.780-0.859)	70.1 (63.1-76.2)	81.0 (75.8-85.3)	76.4 (72.2-80.1)	0.804 (0.758-0.846)
Multi	Multi	81.7 (78.3-84.7)	79.5 (75.1-83.2)	80.8 (78.2-83.2)	0.871 (0.848-0.893)	77.9 (72.7-82.4)	81.4 (78.3-84.2)	80.4 (77.8-82.8)	0.869 (0.842-0.893)

**Figure 6:**
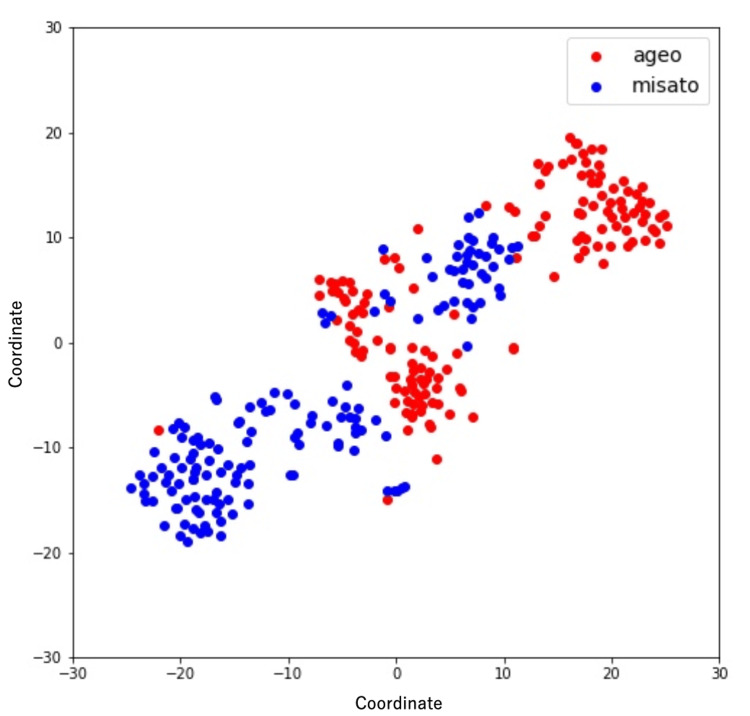
Scatter plots of chest radiograph data reduced to two dimensions using t-SNE Vertical and horizontal axes indicate relative distances between data. t-SNE, t-distributed stochastic neighbor embedding

Additionally, the Misato model had a lower screening performance than the Ageo model, indicating that there were differences in diagnostic performance among facilities. In addition, applying t-SNE to the images from each facility revealed distinct clusters corresponding to each facility. The fact that the points plotted using t-SNE are close to each other means that the data have similar characteristics, and it was suggested that the image data from the same facility has hidden similar characteristics (Figure 6).

**Figure 7:**
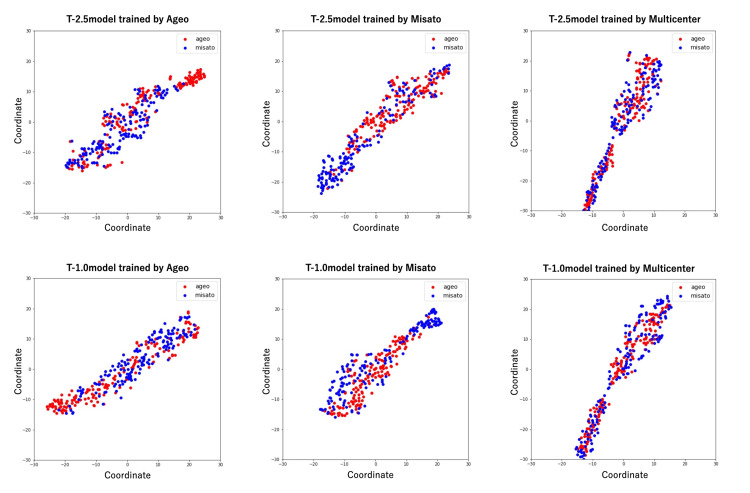
Scatterplot of two-dimensional feature representations extracted by the model using t-SNE Vertical and horizontal axes indicate relative distances between data. t-SNE, t-distributed stochastic neighbor embedding

The t-SNE results of the final convolutional layer’s output from each model also demonstrated cluster formation for each facility in the Ageo and Misato models, suggesting that these models detected facility-specific characteristics (Figure 7). In contrast, no cluster formation was observed in the multicenter model, indicating successful learning that generalized across facility-specific differences.

**Figure 8:**
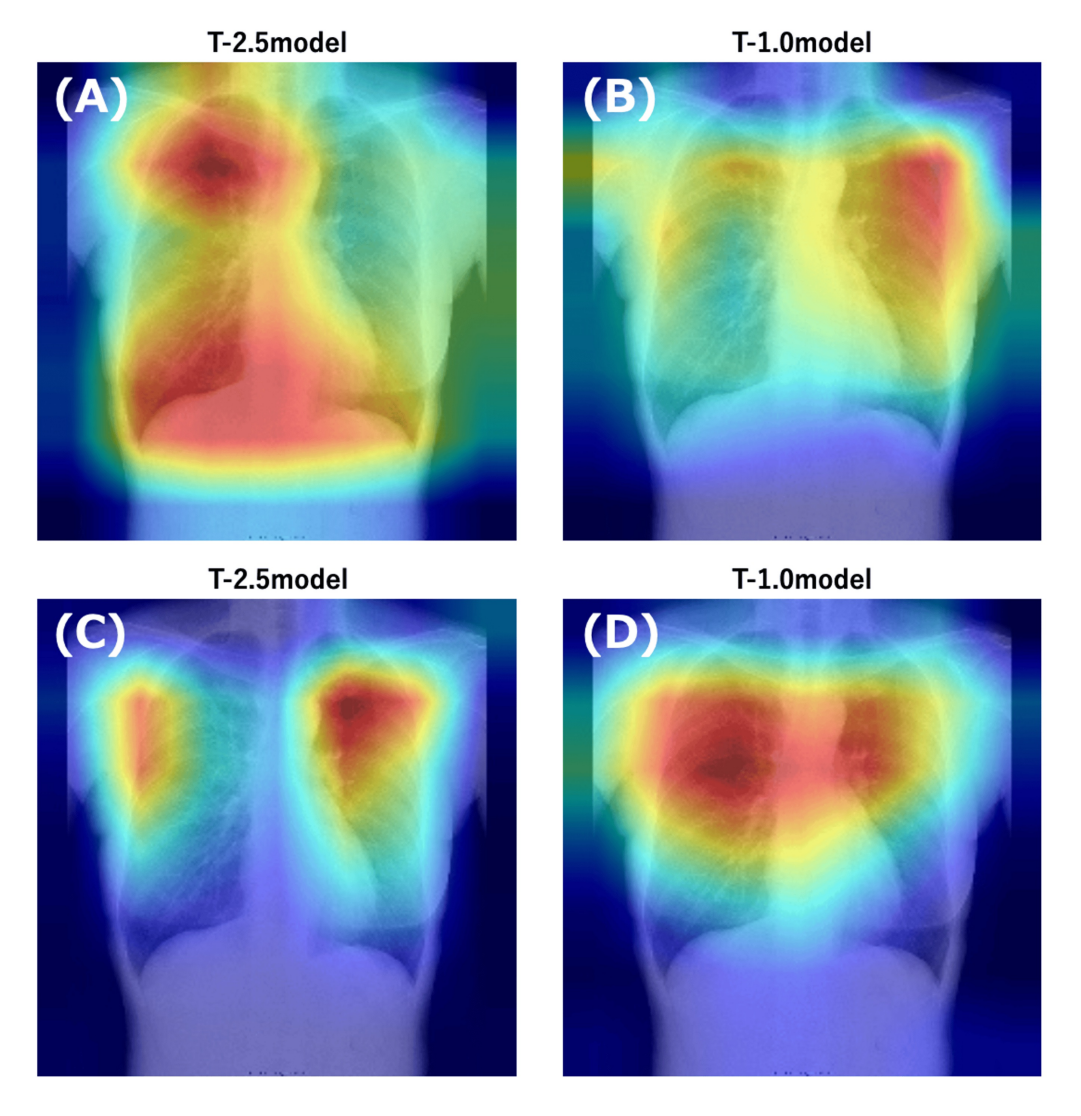
Visualize the diagnostic basis of each model by overlaying the Grad-CAM output on each image (A, B) Grad-CAM highlights the features leading to an osteoporosis diagnosis. (C, D) Grad-CAM highlights the features supporting a normal diagnosis. The superimposed heatmap highlights regions of the medical image that the Grad-CAM technique identified as most influential in the model’s decision-making process. Red areas indicate higher attention, signifying regions that had the strongest positive impact on the prediction, while cooler colors, moving toward blue, represent areas of lower influence. Grad-CAM, gradient-weighted class activation mapping

To visualize the diagnostic basis, a heat map was generated using Grad-CAM. Each model focused on both the spine, which is the target site for DXA, and on various other sites, such as the clavicle, ribs, and lower rib cage (Figure 8). This suggests that the models focus on different regions, rather than on only one specific bone.

The osteoporosis screening model (multicenter model) developed in this study required approximately 1 minute to diagnose 950 test data. Thus, the osteoporosis screening model took only 1 s to diagnose osteoporosis from a single CR.

Subgroup study

To improve the screening performance of external data, we added external data (250, 500, 750, 1000, and 4000 cases) from different facilities to test the Ageo and Misato models. Additionally, the data were randomly selected for the T-score distribution to match that of the sample population.

**Figure 9:**
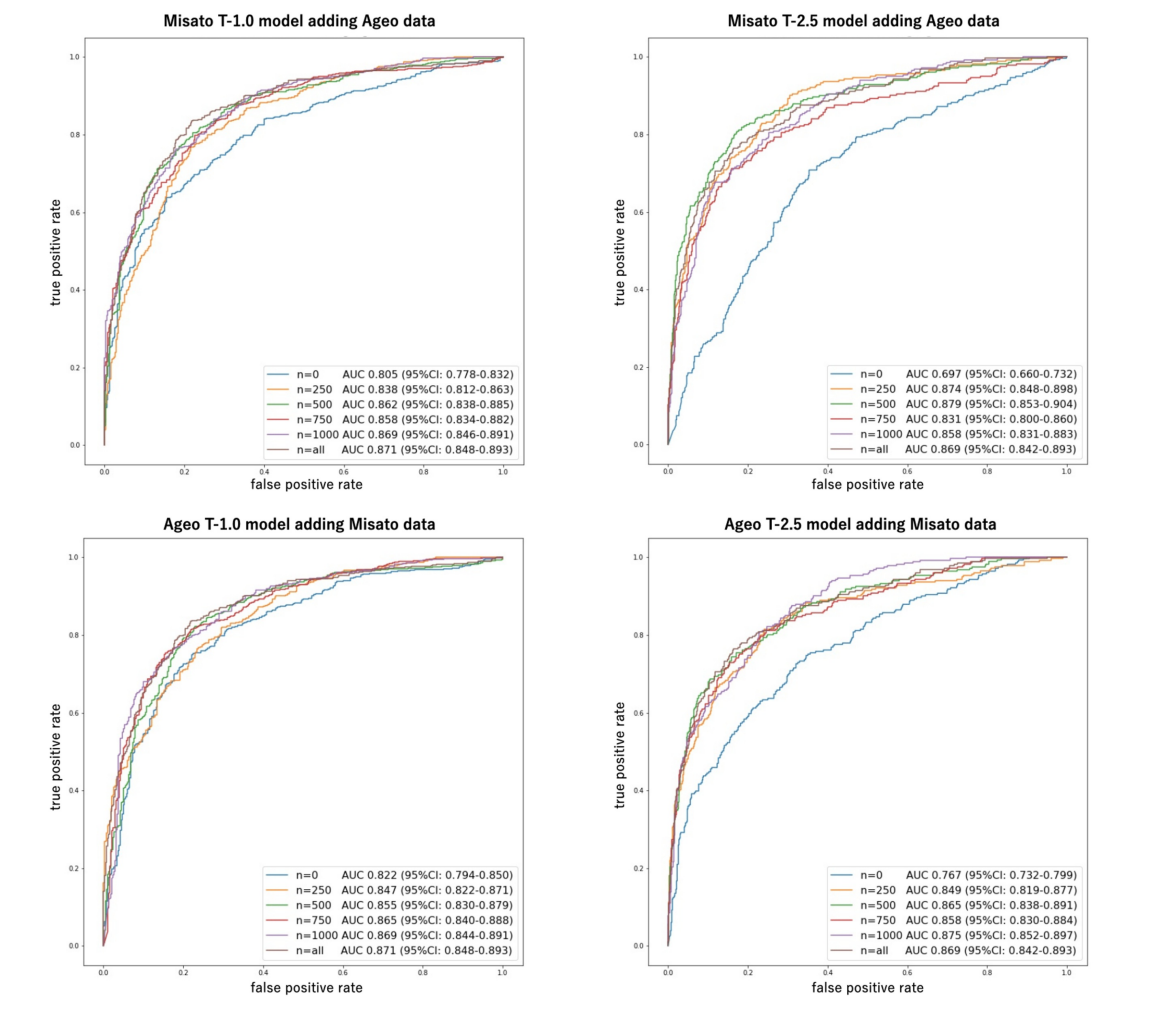
ROC curves for each model trained with 0, 250, 500, 750, 1000, and all external cases added to the training data

Figure 9 shows the ROCs and AUCs for each model, and their detailed performances are described in Table 3 and Table 4. The performance of internal data does not decrease with the addition of external data. The results also suggest that the screening performance for the external test data increases to the same level as that for the internal test data when 500 or more images from other facilities are added (Table 5).

**Table 3:** Model performance of the T-1.0 model with external data added to the training data for each facility

Training data	T-1.0 model					
	Test by Ageo			Test by Misato		
	Specificity	Sensitivity	AUC	Specificity	Sensitivity	AUC
Ageo (all) + Misato (0 data)	82.5 (77.2-86.8)	82.6 (77.5-86.7)	0.901 (0.873-0.926)	81.4 (74.2-87.0)	59.3 (53.7-64.7)	0.743 (0.694-0.791)
Ageo (all) + Misato (250 data)	82.9 (77.6-87.2)	81.8 (76.7-86.0)	0.896 (0.868-0.922)	65.7 (57.5-73.1)	71.1 (65.8-75.9)	0.772 (0.727-0.815)
Ageo (all) + Misato (500 data)	77.5 (71.8-82.3)	84.5 (79.6-88.3)	0.888 (0.859-0.916)	76.4 (68.8-82.7)	79.7 (74.8-83.8)	0.824 (0.780-0.864)
Ageo (all) + Misato (750 data)	85.0 (79.9-89.0)	78.4 (73.1-82.9)	0.894 (0.866-0.921)	72.1 (64.2-78.9)	77.7 (72.7-82.0)	0.815 (0.772-0.856)
Ageo (all) + Misato (1000 data)	88.8 (84.1-92.2)	75.0 (69.4-79.8)	0.906 (0.880-0.931)	63.6 (55.3-71.1)	81.0 (76.2-85.0)	0.807 (0.762-0.850)
Ageo (all) + Misato (2000 data)	83.3 (78.1-87.5)	81.8 (76.7-86.0)	0.913 (0.888-0.936)	68.6 (60.5-75.7)	84.3 (79.8-87.9)	0.832 (0.790-0.870)
Ageo (all) + Misato (3000 data)	86.7 (81.8-90.4)	80.3 (75.1-84.7)	0.906 (0.879-0.930)	66.4 (58.3-73.7)	79.0 (74.1-83.2)	0.804 (0.761-0.844)
Ageo (all) + Misato (4000 data)	84.2 (79.0-88.2)	79.2 (73.9-83.6)	0.910 (0.885-0.933)	70.0 (62.0-77.0)	78.4 (73.4-82.6)	0.823 (0.784-0.861)
Ageo (all) + Misato (all)	85.0 (79.9-89.0)	83.7 (78.8-87.7)	0.908 (0.881-0.932)	70.0 (62.0-77.0)	78.0 (73.1-82.3)	0.821 (0.780-0.859)
Ageo (0 data) + Misato (all)	34.6 (28.9-40.8)	96.2 (93.2-97.9)	0.839 (0.803-0.872)	78.6 (71.1-84.6)	76.4 (71.3-80.8)	0.859 (0.824-0.891)
Ageo (250 data) + Misato (all)	88.8 (84.1-92.2)	69.3 (63.5-74.6)	0.874 (0.842-0.903)	57.1 (48.9-65.0)	84.6 (80.1-88.2)	0.799 (0.755-0.840)
Ageo (500 data) + Misato (all)	86.7 (81.8-90.4)	73.1 (67.5-78.1)	0.888 (0.858-0.915)	65.0 (56.8-72.4)	83.9 (79.4-87.6)	0.821 (0.780-0.860)
Ageo (750 data) + Misato (all)	80.4 (74.9-84.9)	75.4 (69.8-80.2)	0.871 (0.840-0.900)	72.1 (64.2-78.9)	82.3 (77.6-86.2)	0.841 (0.802-0.877)
Ageo (1000 data) + Misato (all)	87.1 (82.2-90.7)	76.5 (71.0-81.2)	0.893 (0.864-0.920)	73.6 (65.7-80.2)	76.1 (71.0-80.5)	0.838 (0.800-0.873)
Ageo (2000 data) + Misato (all)	87.9 (83.2-91.5)	75.0 (69.4-79.8)	0.899 (0.871-0.924)	68.6 (60.5-75.7)	83.9 (79.4-87.6)	0.842 (0.802-0.880)
Ageo (3000 data) + Misato (all)	80.0 (74.5-84.6)	81.4 (76.3-85.7)	0.909 (0.883-0.932)	77.9 (70.3-83.9)	76.7 (71.7-81.1)	0.840 (0.802-0.877)
Ageo (4000 data) + Misato (all)	80.0 (74.5-84.6)	87.5 (83.0-91.0)	0.912 (0.885-0.936)	79.3 (71.8-85.2)	78.7 (73.8-82.9)	0.869 (0.834-0.900)
Ageo (all) + Misato (all)	85.0 (79.9-89.0)	83.7 (78.8-87.7)	0.908 (0.881-0.933)	70.0 (62.0-77.0)	78.0 (73.1-82.3)	0.821 (0.778-0.859)

**Table 4:** Model performance of the T-2.5 model with external data added to the training data for each facility

Training data	T-2.5 model					
	Test by Ageo			Test by Misato		
	Specificity	Sensitivity	AUC	Specificity	Sensitivity	AUC
Ageo (all) + Misato (0 data)	85.9 (82.1-88.9)	76.6 (67.1-84.0)	0.898 (0.862-0.929)	96.1 (93.0-97.9)	28.3 (22.4-35.2)	0.734 (0.684-0.779)
Ageo (all) + Misato (250 data)	90.5 (87.3-93.0)	74.5 (64.8-82.2)	0.915 (0.879-0.945)	77.9 (72.5-82.5)	65.2 (58.2-71.7)	0.775 (0.730-0.821)
Ageo (all) + Misato (500 data)	88.5 (85.1-91.3)	76.6 (67.1-84.0)	0.911 (0.879-0.939)	80.2 (74.9-84.6)	69.0 (62.0-75.2)	0.818 (0.775-0.859)
Ageo (all) + Misato (750 data)	84.1 (80.3-87.4)	84.0 (75.3-90.1)	0.910 (0.879-0.938)	79.1 (73.7-83.6)	70.6 (63.7-76.7)	0.820 (0.779-0.858)
Ageo (all) + Misato (1000 data)	87.8 (84.3-90.6)	68.1 (58.1-76.6)	0.901 (0.869-0.929)	70.5 (64.7-75.8)	73.8 (67.1-79.6)	0.827 (0.788-0.864)
Ageo (all) + Misato (2000 data)	91.0 (87.8-93.4)	68.1 (58.1-76.6)	0.903 (0.870-0.931)	74.4 (68.8-79.4)	71.7 (64.8-77.6)	0.812 (0.770-0.853)
Ageo (all) + Misato (3000 data)	92.2 (89.2-94.4)	70.2 (60.3-78.5)	0.918 (0.890-0.943)	76.4 (70.8-81.1)	71.7 (64.8-77.6)	0.805 (0.760-0.847)
Ageo (all) + Misato (4000 data)	86.3 (82.7-89.3)	86.2 (77.8-91.7)	0.931 (0.906-0.953)	51.6 (45.5-57.6)	81.3 (75.1-86.2)	0.771 (0.727-0.815)
Ageo (all) + Misato (all)	92.4 (89.5-94.6)	71.3 (61.4-79.4)	0.928 (0.902-0.951)	81.0 (75.8-85.3)	70.1 (63.1-76.2)	0.804 (0.758-0.846)
Ageo (0 data) + Misato (all)	21.2 (17.5-25.4)	100.0 (96.1-100.0)	0.781 (0.734-0.825)	73.6 (67.9-78.6)	75.4 (68.8-81.0)	0.806 (0.760-0.848)
Ageo (250 data) + Misato (all)	86.1 (82.4-89.1)	79.8 (70.6-86.7)	0.916 (0.887-0.941)	76.4 (70.8-81.1)	72.7 (65.9-78.6)	0.839 (0.802-0.875)
Ageo (500 data) + Misato (all)	87.1 (83.5-90.0)	79.8 (70.6-86.7)	0.933 (0.909-0.954)	84.1 (79.2-88.1)	72.7 (65.9-78.6)	0.847 (0.807-0.883)
Ageo (750 data) + Misato (all)	83.4 (79.5-86.7)	78.7 (69.4-85.8)	0.894 (0.861-0.924)	72.5 (66.7-77.6)	70.6 (63.7-76.7)	0.781 (0.734-0.826)
Ageo (1000 data) + Misato (all)	86.3 (82.7-89.3)	73.4 (63.7-81.3)	0.906 (0.877-0.932)	60.5 (54.4-66.2)	80.2 (73.9-85.3)	0.807 (0.765-0.848)
Ageo (2000 data) + Misato (all)	83.2 (79.2-86.5)	78.7 (69.4-85.8)	0.890 (0.860-0.918)	62.8 (56.7-68.5)	77.0 (70.5-82.5)	0.800 (0.755-0.841)
Ageo (3000 data) + Misato (all)	89.8 (86.4-92.3)	77.7 (68.2-84.9)	0.923 (0.898-0.947)	82.2 (77.0-86.4)	68.4 (61.5-74.7)	0.819 (0.778-0.858)
Ageo (4000 data) + Misato (all)	87.3 (83.7-90.2)	78.7 (69.4-85.8)	0.914 (0.885-0.940)	71.7 (65.9-76.9)	73.8 (67.1-79.6)	0.817 (0.775-0.857)
Ageo (all) + Misato (all)	92.4 (89.5-94.6)	71.3 (61.4-79.4)	0.928 (0.902-0.951)	81.0 (75.8-85.3)	70.1 (63.1-76.2)	0.804 (0.760-0.847)

**Table 5:** P-value of each model’s performance compared to the multicenter model *P-value <0.05 was considered statistically significant.

	T-1.0				T-2.5		
Model adding external data	Sensitivity	Specificity	AUC		Sensitivity	Specificity	AUC
Ageo full data + Misato 250 data	>0.0001*	0.0375*	0.0103*		0.1875	0.7333	0.1306
Ageo 250 data + Misato full data	0.06	0.3272	0.0099*		0.2828	0.0057*	0.2085
Ageo full data + Misato 500 data	0.7607	0.1753	0.8391		0.6481	0.444	0.3688
Ageo 500 data + Misato full data	0.7171	0.0353*	0.094		0.1898	0.0753	0.5879

## Discussion

Our deep learning-based osteoporosis screening AI was able to diagnose patients with osteoporosis from a single CR. In a previous study [14], the diagnostic performance for osteopenia was AUC 0.70 (95% CI: 0.68-0.72), sensitivity 71.28% (95% CI: 69.01-73.53), and specificity 62.35% (95% CI: 59.94-64.77). For osteoporosis, the diagnostic performance was AUC 0.84 (95% CI: 0.82-0.86), sensitivity 77.27% (95% CI: 74.94-79.36), and specificity 78.58% (95% CI: 76.32-80.55). The performance of our multicenter model was comparable to or better than those previously reported. Additionally, the screening performance on external data across all our models was comparable to or better than that of QUS. We conducted detailed validation of model performance and data visualization to support and guide further improvements.

Our model can be used in various settings, and the use of CR images is highly effective for screening. In previous QUS studies, the AUC for detecting a DXA T-score <-1.0 ranged from 0.666 to 0.739, and for detecting a DXA T-score <-2.5 ranged from 0.706 to 0.758, indicating that the screening performance on external data in all our models was comparable to or better than that of QUS. Because there are significant biases and poor robustness in external facilities [20,21], the robustness of external data is extremely important in medical AI, especially when using examinations performed under various conditions, such as CR. Furthermore, good performance of the T-score 1.0 model is crucial for screening potential osteoporotic patients who have not yet suffered fragility fractures [38,39]. Worldwide, 3.6 billion X-ray examinations are performed annually, with CR accounting for the majority (40%) [40,41]. Our AI can easily accompany the 1.44 billion examinations for osteoporosis screening without additional cost or invasiveness, and its screening effectiveness is expected to be significantly high. Even with the consideration of external robustness, it is likely to be used as a screening test for osteoporosis, although the screening performance still needs improvement.

We also explore approaches for further model improvements in the future. The results of the visualization using t-SNE clearly indicate that medical images contain facility-specific features that we are unaware of and that the model could capture these hidden features. The results of this study support previous claims that differences in images, due to examination equipment, imaging conditions, and facility-specific information, can degrade external robustness [20,21]. Given the scarcity of studies applying t-SNE to medical images, our t-SNE analysis provides a valuable contribution to understanding how image-specific information contributes to this degradation. While some multicenter studies have conducted external validation, the impact of varying imaging equipment types and acquisition parameters across participating facilities has often been overlooked [16]. This detailed investigation into the influence of diverse imaging environments on model robustness, a strength of the present study, represents a significant advancement over prior research. One strategy to address these effects is to calibrate the model for each facility in which it is used. We provide guidance on the number of cases to be added to the training data for calibration. Our subgroup analysis suggests that adding a minimum of 500 external facility cases can effectively calibrate the model. Rather than collecting several thousand cases from a few facilities, our findings indicate that collecting a few hundred cases from many facilities may be more effective in enhancing model robustness. We believe these findings represent an important contribution to addressing the challenge of robustness to external data, which remains a key limitation in many diagnostic imaging AIs.

Furthermore, our screening model could identify osteoporotic changes in various bones and estimate BMD in the lumbar spine and femur. Although the clavicle and ribs are not typically used to evaluate osteoporosis, there are reports of a significant correlation between clavicle cortical thickness and femur BMD [42,43]. Structural changes occur in the ribs with aging [44,45], and there are many other bone sites apart from the spine and femur in which osteoporotic changes occur. The Grad-CAM results and their ability to estimate BMD, including that of the femur, which does not appear on CR images, provide evidence to support these findings.

While this study demonstrates significant potential of AI to diagnose osteoporosis from CR images and offers valuable insights into enhancing model robustness, it is important to acknowledge several limitations that warrant future investigation. First, as a retrospective study, our findings serve as a crucial step toward clinical implementation, but prospective validation in diverse real-world settings is essential to fully confirm long-term clinical applicability and effectiveness. Additionally, the performance could be improved by increasing the number of facilities from which data is retrieved [46,47], and therefore, we hope to increase the number of facilities and the amount of data in the future. However, when introducing the system into clinical practice, we must take measures, such as calibration with facility data and limiting the types of imaging equipment [23,48], to prevent a decline in diagnostic performance. Furthermore, while this study has provided more detailed insights into device variability across different facilities, including the specific CR and DXA equipment detailed in the Methods section, and its potential impact on model performance (e.g., through t-SNE analysis), we acknowledge that fully elucidating the exact causes of performance degradation in external datasets remains challenging. Future studies should aim to quantitatively explore how these specific variations affect model robustness. We also recognize that achieving complete reproducibility in deep learning research remains an ongoing challenge, as subtle variations in computational environments (e.g., software versions beyond major libraries, hardware configurations, random seeds) can influence the exact replication of results, despite our detailed methodological descriptions. Second, we did not perform a secondary analysis after adjusting for data distribution by age group. Subgroup analysis to identify algorithm bias is crucial [23,49,50], and test data that more closely reflect the usage environment may reveal performance closer to that seen in real-world clinical settings [23]. In a prior study [14], only 17.6% of the data were normal (T-score >-1.0); therefore, deviation from the clinical data distribution may have affected performance. A strength of our model is that the dataset is well-balanced and includes relatively young females and males, making it suitable for use in various contexts. In the future, we aim to evaluate and improve the model’s performance across different data distributions through additional validation by age groups. Third, incorporating patient information into the model may improve its performance. In this study, age and sex were excluded from the DICOM data used for training because sex could often be inferred from the images, and age was excluded to prevent overfitting due to its strong correlation with the T-score. However, incorporating information such as responses from the FRAX score questionnaire [51,52] may further enhance model performance [53]. Although this AI currently estimates only BMD, we hope to integrate data for assessing fracture risk in the future, as the essence of osteoporosis lies in the risk of fracture.

We have addressed these limitations and introduced this screening AI into clinical practice to help reduce the number of people suffering from osteoporosis.

## Conclusions

This study developed and extensively validated deep learning models for opportunistic osteoporosis screening using multicenter CR. Our model demonstrated robust performance, comparable to or exceeding existing screening tools, on both internal and external datasets. A key finding is that facility-specific variations, such as imaging equipment and acquisition parameters, can influence model robustness in diverse clinical environments. We provide a practical strategy: recalibrating the model with a minimum of 500 external data points can substantially improve performance, even when facility-specific biases are present. These findings highlight the potential of AI-driven osteoporosis screening from widely accessible CRs as a cost-effective and non-invasive solution. Our research advances the reliable clinical application of deep learning for osteoporosis, emphasizing the importance of rigorous external validation and practical calibration for robust real-world performance.
